# [3 + 2]-Cycloadditions of nitrile ylides after photoactivation of vinyl azides under flow conditions

**DOI:** 10.3762/bjoc.9.201

**Published:** 2013-08-26

**Authors:** Stephan Cludius-Brandt, Lukas Kupracz, Andreas Kirschning

**Affiliations:** 1 Institute of Organic Chemistry, Leibniz University Hannover, Schneiderberg 1b, 30167 Hannover, Germany

**Keywords:** azirines, cycloaddition, flow chemistry, flow reactors, inductive heating, nitrile ylides, photochemistry, vinyl azides

## Abstract

The photodenitrogenation of vinyl azides to 2*H*-azirines by using a photoflow reactor is reported and compared with thermal formation of 2*H*-azirines. Photochemically, the ring of the 2*H*-azirines was opened to yield the nitrile ylides, which underwent a [3 + 2]-cycloaddition with 1,3-dipolarophiles. When diisopropyl azodicarboxylate serves as the dipolarophile, 1,3,4-triazoles become directly accessible starting from the corresponding vinyl azide.

## Introduction

Recently, photochemistry has seen a renaissance despite the fact that under batch conditions specialized reaction vessels are required, in which the light source is placed in the centre of the reaction mixture: Technically this setup is difficult to control for large scale industrial applications because the issue of transferring a substantial amount of heat has to be addressed. On the other hand, photochemistry allows to perform many transformations that are hardly possible under thermal conditions. This includes photocatalytic reactions that have seen an immense interest lately [1].

**Scheme 1:**
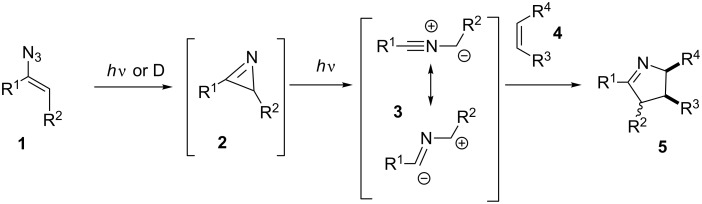
Formation of azirines **2** from vinyl azides **1**, photoinduced ring-opening to the nitrile ylides **3,** and 1,3-dipolar cycloaddition to the pentacyclic *N*-heterocycles **5**.

Nitrile ylides **3** are 1,3-dipoles that have served for the preparation of different five-membered *N*-heterocycles in 1,3-dipolar cycloaddition reactions. They are commonly formed through three routes which are a) the addition of electrophilic carbenes to nitriles, b) the dehydrochlorination of imidoyl chlorides, and c) the photochemical ring opening of strained 2*H*-azirines **2** [2–5]. The latter route can be initiated by the photoinduced activation of vinyl azides **1**, which gives rise to 2*H*-azirines **2** via vinyl nitrenes after the loss of molecular nitrogen and subsequent ring-opening under photochemical conditions to provide the nitrile ylides **3** (Scheme 1). For recent examples for the use of azirines in organic syntheses please refer to [6–15]. Recently, the Seeberger group has published a flow protocol on the photochemical degradation of aryl azides and the subsequent formation of 3*H*-azepinones [16].

With the emergence of continuous processes involving miniaturized flow reactors in organic-chemistry laboratories, photochemistry has found a wider interest in the chemical community [17–18]. Particularly large-scale photochemical syntheses can simply be achieved by numbering-up miniaturized flow reactors in a parallel set-up. Uniform irradiation can be guaranteed when the penetration depth of light is kept small (100–1000 µm). Furthermore, the production rate of a photochemical flow process can be controlled by varying the irradiation power, or by increasing or decreasing of the flow rate. Finally, miniaturized flow reactors have high heat-transfer coefficients so that the cooling of the photochemical process can efficiently be achieved.

These facts led us to initiate an investigation on the photochemical activation of vinyl azides and the trapping of the intermediate nitrile ylides **3** [19] by different dipolarophiles exploiting the advantages of photo flow-chemistry [20–21]. Here, we report on the first photochemical transformation of vinyl azides to pyrrole derivatives under continuous-flow conditions.

**Scheme 2:**
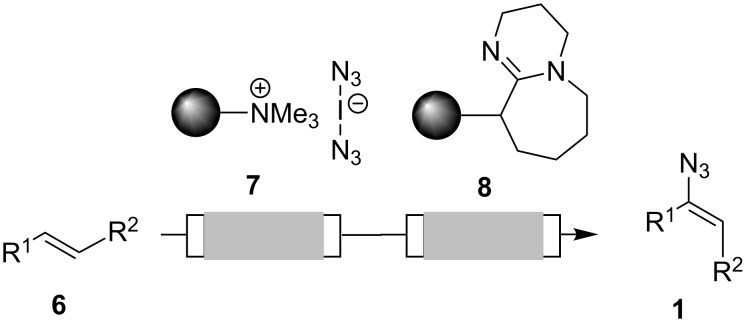
Solid-phase assisted synthesis of vinyl azides **1** from alkenes **6** under flow conditions [9].

Only recently, we reported the two-step preparation of vinyl azides **1** in mircrostructured flow reactors starting from alkenes **6**, using the solid-phase bound iodine azide transfer-reagent **7** followed by HI elimination using immobilized DBU as fixed bed material (Scheme 2) [9,22–23]. All vinyl azides used in this report were prepared by azido-iodination of the corresponding alkenes followed by DBU-mediated HI elimination (for details see the Supporting Information File 1).

## Results and Discussion

**Scheme 3:**
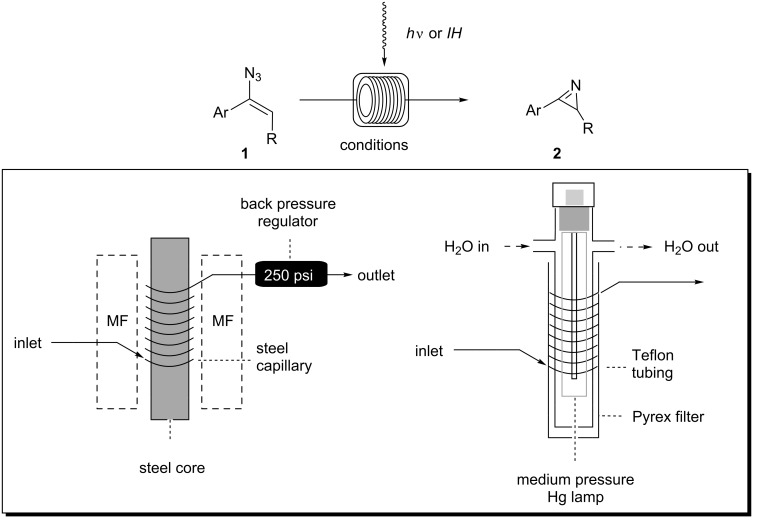
Schematic presentation of the flow set-up for the synthesis of *2H-*azirines **2** under inductive heating (*IH*, left) and photochemical (*h*ν, right) conditions.

As the generation of azirines **2** can be conducted under thermal as well as under photochemical conditions, we first evaluated both processes with respect to their suitability under flow conditions (Scheme 3). The thermal reaction was studied in the presence of an external oscillating magnetic field of medium frequency (15–25 kHz). The best reactor set-up for inducing heat in a medium frequency field was found to be a steel capillary reactor (volume: 1.0 mL, inner diameter = 1.0 mm) with a steel core, which is encased by the inductor. An internal pressure of at least 250 psi allows transformations well above the boiling point of the solvent, and this was secured by placing a backpressure regulator behind the flow system. In contrast, the photochemical flow-reactor was composed of a Teflon (FEP) tubing (volume: 3.0 mL, inner diameter = 0.75 mm) and a Pyrex filter. These were placed onto the water-cooled quartz immersion well (type UV-RS-1, Heraeus) equipped with a medium-pressure mercury lamp (type TQ 150, λ = 190–600 nm). The reaction mixture was fed into the tubing by using a pump and collected in a flask after having passed through the reactor.

In essence 2*H-*azirines can be prepared continuously in good yields under thermal as well as under photochemical conditions in appropriate flow reactor devices (Table 1). Complete conversion was achieved at 190 °C after 1 min in dichloromethane. At higher temperatures as well as at reduced flow rates the amount of decomposition products increased. The photochemical transformation required longer reaction times, but the products were formed under thermally mild conditions in improved yields and with higher purity. Therefore, we decided to continue our studies with the photochemical flow-reactor and to extend these studies to the photoinduced nitrile ylide formation and the 1,3-dipolar cycloaddition. We initially chose to photolyze methyl 4-(1-azidovinyl)benzoate (**1a**) in the presence of acrylonitrile (**4a**) (Table 2). A solution of **1a** and **4a** in the respective solvent was passed through the photochemical flow-reactor with 5.5 mL volume and a pyrex filter.

**Table 1:** Continuous synthesis of *2H-*azirines **2** under inductive heating and photochemical conditions. The experiments were conducted at a concentration of 0.05 M.

entry	product^a^	isolated yield (*h*ν) [%]^b,c^	isolated yield (*IH*) [%]^c,d^

1	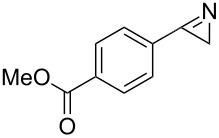 **2a**	97	82
2	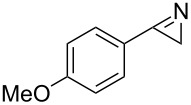 **2b**	90	79
3	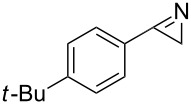 **2d**	92	72
4	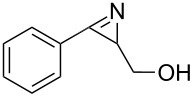 **2g**	95	42^e^

^a^Precursor vinyl azides and 2*H*-azirines are found in Scheme 4; ^b^photo flow-conditions: toluene, 10 min (residence time), rt; ^c^isolated yields are given; ^d^inductive heating conditions: CH_2_Cl_2_, 1 min (residence time), 190 °C; ^e^although the transformation was very rapid, we encountered substantial decomposition under thermal conditions.

**Table 2:** Optimization of the photolysis of vinyl azide **1a** and trapping of nitrile ylide with acrylonitrile **4a** under flow conditions.

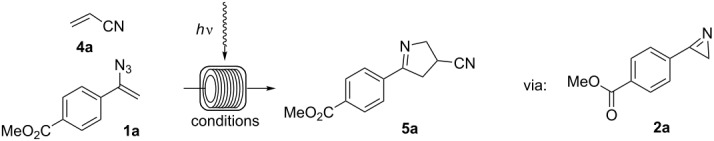					

entry	concentration of **1a** [M]	ratio (**1a**:**4a**)	solvent	flow rate [mL/min]	isolated yield [%] of **5a**

1	0.025	1:10	toluene	0.05	-
2	0.025	1:10	benzene	0.05	-
3	0.025	1:10	CH_3_CN	0.05	46
4	0.025	1:10	CH_3_CN	0.1	53
5	0.012	1:10	CH_3_CN	0.05	82
6	0.012	1:10	CH_3_CN	0.1	74
7	0.012	1:10	CH_3_CN	0.2	68
8	0.05	1:10	CH_3_CN	0.05	96
9	0.05	1:5	CH_3_CN	0.05	71
10	0.05	1:2	CH_3_CN	0.05	65

Test reactions conducted either in benzene or in toluene resulted exclusively in the formation of the corresponding 2*H*-azirine **2a** in yields up to 95%, while no formation of the cycloaddition product was encountered (Table 2; entries 1 and 2). **2a** could easily be identified by the signal at 1.88 ppm in the ^1^H NMR spectrum, which is characteristic for the methylene group of the newly formed 3-membered ring. This signal corresponds to the carbon signal at 20.2 ppm in the ^13^C NMR spectrum. In contrast, acetonitrile turned out to be the solvent of choice and methyl 4-(4-cyano-4,5-dihydro-3*H*-pyrrol-2-yl)benzoate (**5a**) was isolated in 46% yield (Table 2, entry 3). By optimizing the reaction conditions with respect to concentration, flow rate, and ratio of starting materials (Table 2, entries 4–10), we found that a concentration of 0.05 mol/L for azide **1a** and a flow rate of 0.05 mL/min in the presence of a tenfold access of **4a** provided the cycloaddition product **5a** in 96% yield as a single regioisomer (Table 2, entry 8). Remarkably, after removal of the solvent under reduced pressure it was not necessary to further purify the product.

**Scheme 4:**
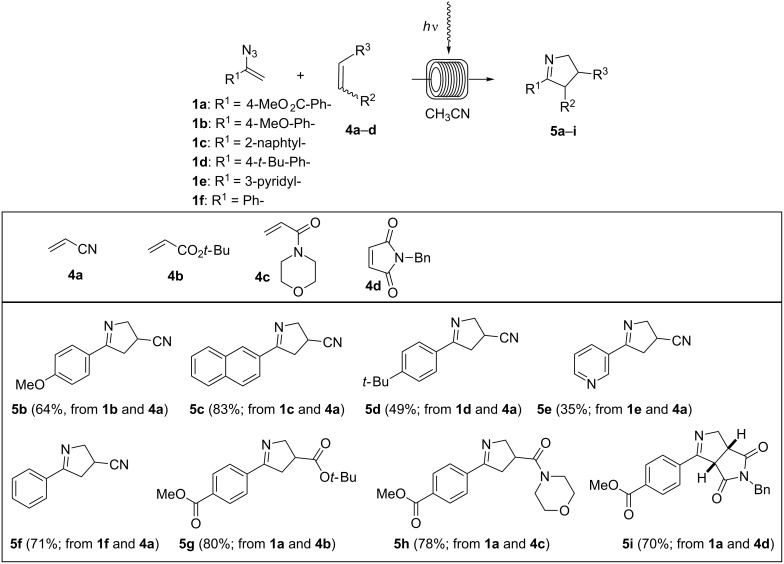
Photoinduced cycloadditions of vinyl azides **1a**–**f** and electron-deficient alkenes **4a**–**d**. All experiments were conducted at room temperature in a photochemical flow-reactor (see above) using Teflon (FEP) tubing (volume: 5.5 mL, inner diameter = 0.75 mm) at a concentration of 0.05 M in CH_3_CN; isolated yields are given.

Next the scope of the photo-induced 1,3-dipolar cycloaddition was examined. With the optimized flow-protocol in hand we were able to synthesize a variety of dihydropyrroles (**5a**–**5i**) (Scheme 4). The electronic properties of the aromatic ring, which depend on the substituents have no prinicipal influence on the outcome of this cascade reaction. Only the pyridyl substituent in vinyl azide **1e** provided dihydropyrrole **5e** in unsatisfactory yield. The relative stereochemistry of **5i** was determined by comparison with literature data [24].

**Scheme 5:**
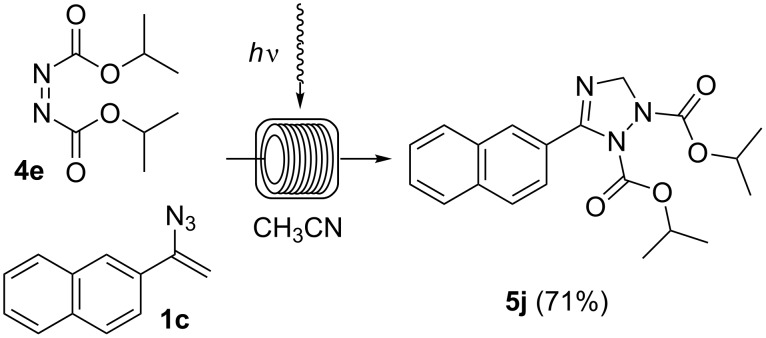
Photoinduced cycloaddtion of vinyl azide **1c** and diisopropyl azodicarboxylate (**4e**). The experiment was conducted at room temperature in a photochemical flow-reactor (see above) using Teflon (FEP) tubing (volume: 5.5 mL, inner diameter = 0.75 mm) at a concentration of 0.05 M in CH_3_CN.

To our delight, this flow protocol also allowed us to prepare 2,3-dihydro-1*H*-1,2,4-triazole **5j** in good yield using diisopropyl azodicarboxylate (DIAD, **4e**) as the dipolarophile (Scheme 5).

**Scheme 6:**
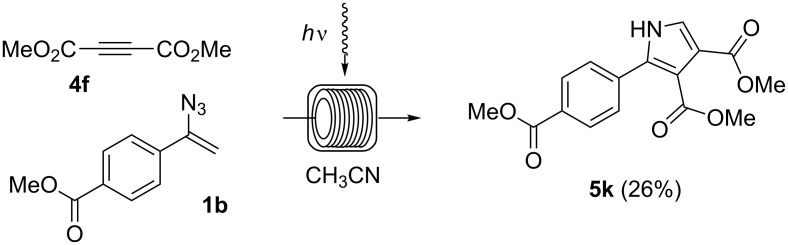
Photoinduced cycloaddtion of vinyl azide **1b** and alkyne **4f**. The experiment was conducted at room temperature in a photochemical flow-reactor (see above) using Teflon (FEP) tubing (volume: 5.5 mL, inner diameter = 0.75 mm) at a concentration of 0.05 M in CH_3_CN.

Additionally, we found that even electron-deficient alkynes such as **4f** can serve as dipolarophiles in these reactions (Scheme 6). However, the resulting pyrrole **5k** could only be isolated in 26% yield.

**Scheme 7:**
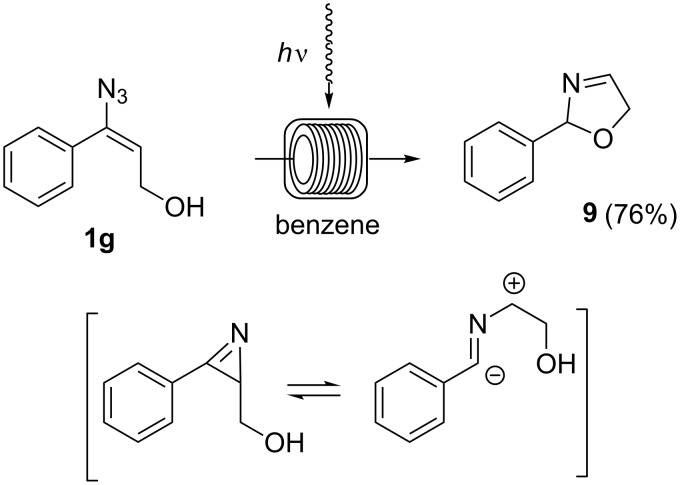
Formation of 2,5-dihydrooxazole **9** starting from vinyl azide **1g** under flow conditions (*c* = 0.01 M, flow rate = 0.02 mL/min).

Alternatively, the in-situ generated nitrile ylide can be trapped intramolecularly by a nucleophile such as a hydroxy group [25]. This is demonstrated by the photochemical degradation of vinyl azide **1g** which yielded 2,5-dihydrooxazole **9** in 76% yield (*c* = 0.01 M, flow rate = 0.02 mL/min) under flow conditions (Scheme 7). In this case, benzene turned out to be the solvent of choice.

## Conclusion

In summary, we developed a protocol for the one-step photochemical formation of dihydropyrroles under flow conditions starting from aromatic vinyl azides and activated alkenes. This transformation was achieved with a photochemical flow reactor and most likely proceeds via the respective 2*H*-azirines by photoinduced in-situ formation and subsequent heterolytic ring opening. The resulting 1,3-dipole is trapped directly with electron-deficient alkenes to form the [2 + 3] cycloaddition products. With this method, we were able to prepare a variety of dihydropyrroles. The electronic properties of the aromatic ring were of little importance for the principal outcome of the reaction. Notable, azodicarboxylates and electron deficient alkynes were employed for the first time which provided a 1,2,4-triazole and a pyrrole, respectively. Future work should cover a further generalization of this flow protocol along with telescoping it with vinyl azide formation.

## Supporting Information

File 1 Descriptions on the synthesis and analyses of vinyl azides and as well as on cycloaddition products.

## References

[1] Griesbeck A G, Steinwäscher J, Reckenthäler M, Uhlig J (2013). Res Chem Intermed.

[2] Padwa A (2010). Adv Heterocycl Chem.

[3] Palacios F, de Retana A M O, de Marigorta E M, de los Santos J M (2002). Org Prep Proced Int.

[4] Palacios F, de Retana A M O, de Marigorta E M, de los Santos J M (2001). Eur J Org Chem.

[5] Heimgartner H (1991). Angew Chem, Int Ed.

[6] Loy N S Y, Singh A, Xu X, Park C-M (2013). Angew Chem, Int Ed.

[7] Khlebnikov A F, Novikov M S, Pakalnis V V, Yufit D S (2011). J Org Chem.

[8] Palacios F, de Retana A M O, del Burgo A V (2011). J Org Chem.

[9] Kupracz L, Hartwig J, Wegner J, Ceylan S, Kirschning A (2011). Beilstein J Org Chem.

[10] Candito D A, Lautens M (2010). Org Lett.

[11] Novikov M S, Amer A A, Khlebnikov A F (2006). Tetrahedron Lett.

[12] Alves M J, Fortes A G, Costa F T (2006). Tetrahedron.

[13] Palacios F, de Retana A M O, Gil J I, Alonso J M (2004). Tetrahedron.

[14] Timén A S, Somfai P (2003). J Org Chem.

[15] Pinho e Melo T M V D, Cardoso A L, Gomes C S B, Rocha Gonsalves A M d’A (2003). Tetrahedron Lett.

[16] Bou-Hamdan F R, Lévesque F, O'Brien A G, Seeberger P H (2011). Beilstein J Org Chem.

[17] Oelgemöller M, Shvydkiv O (2011). Molecules.

[18] Matsushita Y, Ichimura T, Ohba N, Kumada S, Sakeda K, Suzuki T, Tanibata H, Murata T (2007). Pure Appl Chem.

[19] Escolano C, Duque M D, Vázquez S (2007). Curr Org Chem.

[20] Knowles J P, Elliott L D, Booker-Milburn K I (2012). Beilstein J Org Chem.

[21] Hook B D A, Dohle W, Hirst P R, Pickworth M, Berry M B, Booker-Milburn K I (2005). J Org Chem.

[22] Kirschning A, Hashem Md A, Monenschein H, Rose L, Schöning K-U (1999). J Org Chem.

[23] Kirschning A, Monenschein H, Schmeck C (1999). Angew Chem, Int Ed.

[24] Tsuge O, Ueno K, Kanemasa S, Yorozu K (1986). Bull Chem Soc Jpn.

[25] Padwa A, Rasmussen J K, Tremper A (1976). J Am Chem Soc.

