# Supplementary material for: [3 + 2]-Cycloadditions of nitrile ylides after photoactivation of vinyl azides under flow conditions
Source: Beilstein J Org Chem. 2013 Aug 26;9:1745–50. doi: 10.3762/bjoc.9.201 (PMC3778330; doi:10.3762/bjoc.9.201)
Supplement: File 1 — Descriptions on the synthesis and analyses of vinyl azides and as well as on cycloaddition products. [file Beilstein_J_Org_Chem-09-1745-s001.pdf]

Supporting Information

for

[3 + 2]-Cycloadditions of nitrile ylides after photoactivation of vinyl azides under flow conditions

Stephan Cludius-Brandt, Lukas Kupracz and Andreas Kirschning*

Address: Institute of Organic Chemistry, Leibniz University Hannover, Schneiderberg 1b, 30167 Hannover, Germany

Email: Andreas Kirschning - andreas.kirschning@oci.uni-hannover.de

* Corresponding author

Descriptions on the synthesis and analyses of vinyl azides and as well as on cycloaddition products

Experimental

1. General

NMR spectra were recorded on a Bruker AV400 spectrometer at 400 MHz (^{1}H NMR) and at 100 MHz (^{13}C NMR) in $CDCl_3$. ^{1}H NMR and ^{13}C NMR chemical shifts (δ) are reported in parts per million (ppm) relative to residual proton signals in $CDCl_3$ (δ = 7.26, 77.16). Coupling constants (*J*) are reported in Hertz (Hz) and refer to apparent multiplicities. The following abbreviations are used for the multiplicities: s singlet, brs broad singlet, d doublet, t triplet, q quartet, qn quintet, sex sextet, sept septet, m multiplet. Mass spectra (EI) were obtained at 70 eV with a Finnigan Mat 312 or (ESI) with a Q-Tof Premier (Waters). Melting points were determined in open glass capillaries with an OptiMelt melting point apparatus and are uncorrected. Thin layer chromatography (TLC) was carried out on aluminum supported plates with a layer thickness of 0.25 mm (Merck TLC silica gel 60 F_{254}) and the spots were visualised with UV light at 254 nm or by staining with H_2SO_4/4-methoxybenzaldehyde in ethanol. Flash column chromatography was performed on silica gel 60 (particle size 0.040–0.063 mm). Starting materials were purchased from known commercial sources (Sigma-Aldrich, Acros Organics) and were used as received.

The photochemical flow-reactor consisted of a medium pressure mercury lamp (TQ 150, 190–600 nm) equipped with a Pyrex filter. Teflon (FEP) tubing (inner diameter: 0.75 mm, volume: 5.5 mL) was wrapped around the Pyrex filter and connected to a syringe pump (TSE Systems) on the inlet and a collection flask on the outlet side (Figure S1).

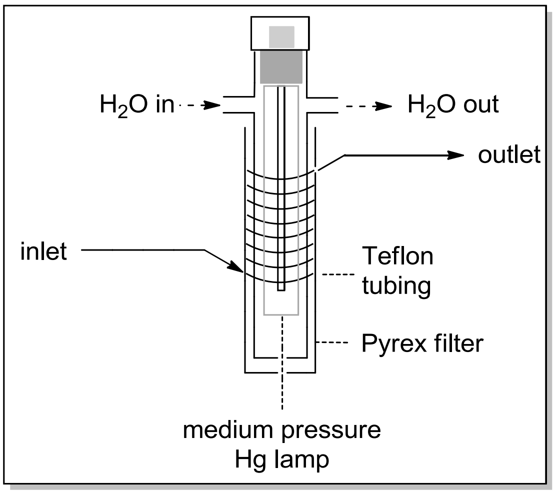

Figure S1: Schematic presentation of the photochemical flow-reactor.

The inductive heating flow-reactor consisted of a steel coil reactor (inner diameter: 1.0 mm, volume: 1.0 mL) that was incased into an inductor (designed and manufactured by IFF GmbH). The heating of the steel coil was achieved by the oscillating electromagnetic field generated in the inductor. The reactor was connected to the pump (Knauer GmbH) and at the outlet a collecting flask was positioned. A system pressure of at least 250 psi was adjusted by implementing a backpressure regulator (Upchurch Scientific) behind the reactor. The temperature was measured on the reactor surface using an IR pyrometer LaserSight model (optris GmbH).

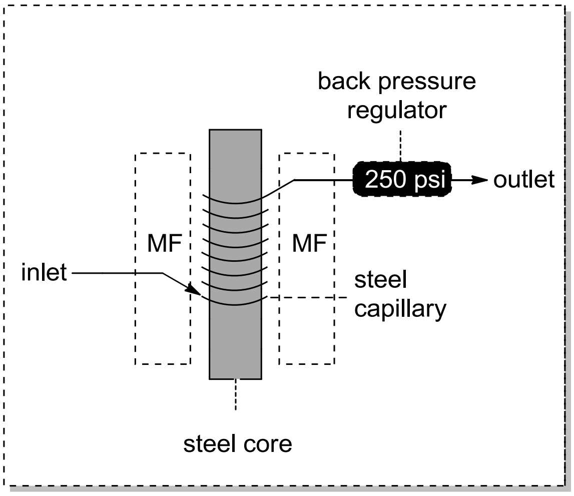

Figure S2: Schematic presentation of the inductive heating flow-reactor.

2. Preparation of vinyl azides

2.1 General considerations

For the formation of vinyl azides **2** starting from alkenes **1**, iodine azide transfer reagent **A** was used (Scheme S1). Chemically polymer-bound bis(azido)iodate(I) **A** behaves like iodine azide but, in contrast it is not explosive and storable for weeks. The two-step procedure consists of a 1,2-azidoiodination reaction of the respective alkenes and subsequent elimination by DBU to form the desired vinyl azides **2**. The good regioselectivity is caused by the generation of the more stable carbenium ion after the elctrophilic attack of the iodonium species on the double bond. The synthesised vinyl azides are depicted in Scheme S1. We could show that this two-step synthesis of vinyl azides is also possible under continuous flow conditions.

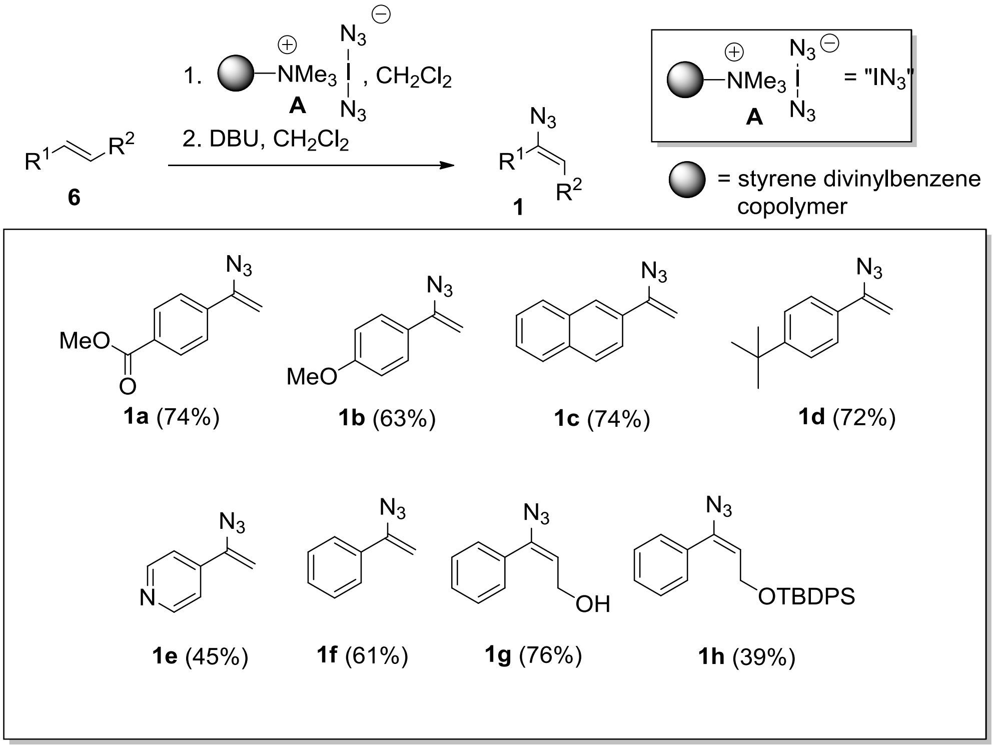

Scheme S1: Two-step formation of vinyl azides **1a**–**1g**. Vinyl azide **1h** was prepared from alcohol **1g** by a standard *O*-silylation protocol.

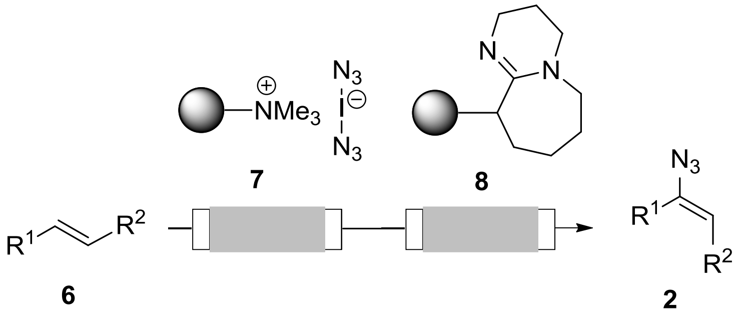

Scheme S2. Solid-phase assisted synthesis of vinyl azides **2** from alkenes **6** under flow conditions.

2 .2 General procedure for the formation of vinyl azides

Polymer-bound iodate(I) complex **A** (12 mmol) was added to a solution of the respective alkene (3 mmol) in dry CH_2Cl_2 (60 mL), the flask was covered with aluminium foil to protect from light and the suspension was shaken at 150 rpm for 24 h. The mixture was filtered and the polymer was washed with CH_2Cl_2 (3 × 20 mL). After evaporation of the solvent the crude azido iodination product was solved in CH_2Cl_2 (30 mL), DBU (6 mmol) was added and the solution was stirred at rt for 4 h. The reaction mixture was diluted with of H_2O (30 mL) and the aqueous phase was extracted with CH_2Cl_2 (3 × 10 mL). The combined organic layers were washed with brine and then dried over $MgSO_4$. After filtration and evaporation of the solvent the crude vinyl azide was purified by flash column chromatography (petroleum ether/ethyl acetate = 9/1).

Methyl 4-(1-azidovinyl)benzoate (1a)

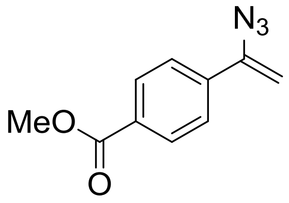

Compound **1a** was prepared in 74% yield (450 mg, 2.2 mmol) from the corresponding alkene as colourless crystals.

MP: 81.9 °C (MP: 84–85 °C [S1])

^{1}H NMR (400 MHz, $CDCl_3$): δ = 8.03 (d, *J* = 8.8 Hz, 2H), 7.64 (d, *J* = 8.8 Hz, 2H), 5.58 (d, *J* = 2.6 Hz, 1H), 5.08 (d, *J* = 2.6 Hz, 1H), 3.94 (s, 3H).

^{13}C NMR (100 MHz, $CDCl_3$): δ = 166.5, 144.2, 138.3, 130.5, 129.7, 125.4, 99.6, 52.2.

HRMS *m/z* (ESI): calcd. $C_{10}H_9N_3O_2$ ($[M - N_2 + H]^+$): 176.0712; found: 176.0788.

All data were in accordance with published data [S1].

1-(1-Azidovinyl)-4-methoxybenzene (1b)

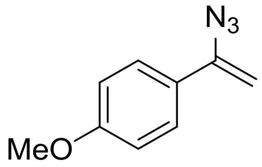

Compound **1b** was prepared in 63% yield (411 mg, 2.3 mmol) from the corresponding alkene as a colourless solid.

MP: 38 °C (MP: 39–40 °C [S2])

^{1}H NMR (400 MHz, $CDCl_3$): δ = 7.51 (d, *J* = 8.9 Hz, 2H), 6.89 (d, *J* = 8.9 Hz, 2H), 5.33 (d, *J* = 2.4 Hz, 1H), 4.87 (d, *J* = 2.4 Hz, 1H), 3.84 (s, 3H).

^{13}C NMR (100 MHz, $CDCl_3$): δ = 160.4, 144.8, 126.9, 113.7, 96.1, 55.3.

HRMS *m/z* (ESI): calcd. $C_9H_{10}N_3O^+$ ($[M + H]^+$): 176.0824; found: 176.0825.

All data were in accordance with published data [S3].

2-(1-Azidovinyl)naphthalene (1c)

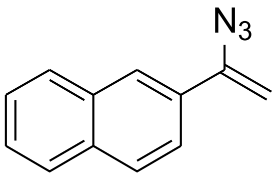

Compound **1c** was prepared in 74% yield (468 mg, 24.0 mmol) from the corresponding alkene as a pale yellow solid.

MP: 56 °C (MP: 56–58 °C [S1])

^{1}H NMR (400 MHz, $CDCl_3$): δ = 8.07 (s, 1H), 7.90-7.81 (m, 3H), 7.68 (dd, *J* = 8.6, 1.9 Hz, 1H), 7.54-7.49 (m, 2H), 5.61 (d, *J* = 2.7 Hz, 1H), 5.08 (d, *J* = 2.7 Hz, 1H).

^{13}C NMR (100 MHz, $CDCl_3$): δ = 145.0, 133.5, 133.1, 131.5, 128.6, 128.2, 127.6, 126.6, 126.5, 125.0, 123.1, 98.3.

HRMS *m/z* (ESI): calcd. $C_{12}H_9N_3^+$ ($[M + H]^+$): 196.0875; found: 196.0871.

All data were in accordance with published data [S3].

1-*tert*-Butyl-4-(1-azidovinyl)benzene (1d)

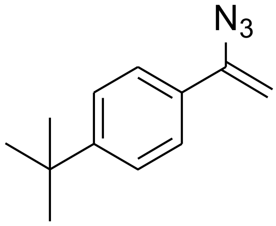

Compound **1d** was prepared in 72% yield (450 mg, 2.2 mmol) from the corresponding alkene as a yellow oil.

^{1}H NMR (400 MHz, $CDCl_3$): δ = 7.45 (d, *J* = 9.0 Hz, 2H), 7.42 (d, *J* = 9.0 Hz, 2H), 5.44 (d, *J* = 2.0 Hz, 1H), 4.96 (d, *J* = 2.0 Hz, 1H), 1.38 (s, 9H).

^{13}C NMR (100 MHz, $CDCl_3$): δ = 152.4, 145.0, 131.5, 125.9, 125.3, 97.4, 34.7, 31.3.

HRMS *m/z* (ESI): calcd. $C_{12}H_{15}N_3^+$ ($[M + H]^+$): 202.1344; found: 202.1348.

All data were in accordance with published data [S3].

4-(1-Azidovinyl)pyridine (1e)

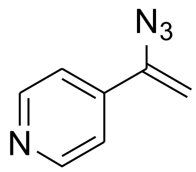

Compound **1e** was prepared in 45% yield (231 mg, 1.6 mmol) from the corresponding alkene as a yellow solid.

MP: 34 °C (MP: 34 °C [S3])

^{1}H NMR (400 MHz, $CDCl_3$): δ = 8.62 (d, *J* = 6.1 Hz, 2H), 7.45 (d, *J* = 6.1 Hz, 2H), 5.69 (d, *J* = 2.7 Hz, 1H), 5.15 (d, *J* = 2.7 Hz, 1H).

^{13}C NMR (100 MHz, $CDCl_3$): δ = 150.2, 143.0, 141.4, 119.5, 100.7.

HRMS *m/z* (ESI): calcd. $C_7H_7N_4^+$ ($[M + H]^+$): 147.0671; found: 147.0670.

All data were in accordance with published data [S3].

1-(1-Azidovinyl)benzene (1f)

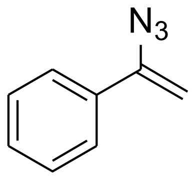

Compound **1f** was prepared in 61% yield (420 mg, 2.9 mmol) from the corresponding alkene as a yellow oil.

^{1}H NMR (400 MHz, $CDCl_3$): δ = 7.58 (m, 2H), 7.38 (m, 3H), 5.45 (d, *J* = 2.4 Hz, 1H), 4.98 (d, *J* = 2.4 Hz, 1H).

^{13}C NMR (100 MHz, $CDCl_3$): δ = 145.0, 134.2, 129.1, 128.4, 125.5, 98.0.

All data were in accordance with published data [S3].

(E)-3-Azido-3-phenylprop-2-en-1-ol (1g)

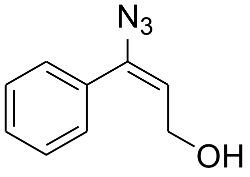

Compound **1g** was prepared in 76% yield (910 mg, 5.2 mmol) from the corresponding alkene as a colourless oil.

^{1}H NMR (400 MHz, $CDCl_3$): δ = 7.44-7.41 (m, 3H), 7.36-7.32 (m, 2H), 5.63 (t, J = 7.5 Hz, 1H), 4.16 (d, J = 7.5 Hz, 2H).

^{13}C NMR (100 MHz, $CDCl_3$): δ = 129.8, 129.5, 129.3, 128.7, 115.2, 59.4.

HRMS *m/z* (ESI): calcd. $C_9H_{10}N_3O^+$ ($[M + H]^+$): 176.0824; found: 176.0821.

All data were in accordance with published data [S3].

TBDPS-protection of 1g

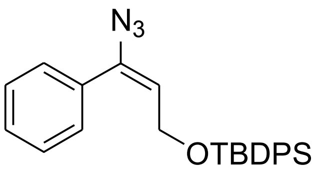

To a solution of **1g** (465 mg, 2.6 mmol, 1 equiv) in CH_2Cl_2 (20 mL) imidazole (218 mg, 3.2 mmol, 1.2 equiv) and TBDPSCl (880 mg, 3.2 mmol, 1.2 equiv) were added and the resulting solution was stirred at room temperature over night. The reaction mixture was then diluted with ether and washed with water (3 × 10 mL) and brine. The organic layer was dried over Na_2SO_4. After

evaporation of the solvents the crude product was purified by flash column chromatography (petroleum ether) to give TBDPS-protected alcohol **1h** as a colourless oil (420 mg, 1.0 mmol, 39%).

^{1}H NMR (400 MHz, $CDCl_3$): δ = 7.65-7.62 (m, 4H), 7.42-7.29 (m, 9H), 7.26-7.21 (m, 2H), 5.59 (d, *J* = 7.3 Hz, 1H), 4.22 (d, *J* = 7.3 Hz, 2H), 1.06 (s, 9H).

^{13}C NMR (100 MHz, $CDCl_3$): δ = 140.1, 135.5, 133.5, 129.6, 129.4 129.0, 128.5, 128.3, 127.6, 127.5, 115.8, 60.5, 26.8, 19.1.

All data were in accordance with published data [S4].

3. General procedure for the preparation of *2H-* azirines under inductive heating conditions

The inductively heatable flow-reactor (void volume = 1 mL) was connected to the pump and at the outlet a backpressure regulator (250 psi) was incorporated into the flow system. The reactor was flushed with CH_2Cl_2 (flow rate = 1.0 mL/min), and the temperature was adjusted to 190 °C by regulating the PWM (pulse-width modulation). After 2 min, a solution of the corresponding vinyl azide (0.05 M) in CH_2Cl_2 was pumped through the reactor at a flow rate of 1.0 mL/min. The crude 2*H*-azirines were obtained after removal of the solvent under reduced pressure.

4. General procedure for the photoinduced preparation of *2H*-azirine under flow conditions

The photo reactor (volume: 3 mL; details see above) was connected to a syringe pump and at the outlet a collection flask was located. A syringe (ø 13.5 mm) was filled with a solution of the respective vinyl azide **1** (0.1 mmol in toluene (1 mL) and the solution was pumped through the reactor (flow rate: 0.3 mL/min). Washing with toluene was continued until no product was detected at the outlet (monitored by TLC). After evaporation of the solvent, the 2*H*-azirine **2** was obtained in good purity.

Methyl 4-(2*H*-azirin-3-yl)benzoate (2a)

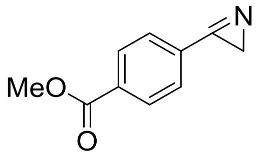

Compound **2a** was prepared in 97% yield (25 mg, 0.14 mmol) from the corresponding vinyl azide as yellow oil.

^{1}H NMR (400 MHz, $CDCl_3$): δ = 8.25 (d, *J* = 8.8 Hz, 2H), 8.01 (d, *J* = 8.8 Hz, 2H), 4.00 (s, 3H), 1.88 (s, 2H).

^{13}C NMR (100 MHz, $CDCl_3$): δ =165.9, 135.4, 130.2, 129.4, 52.6, 20.3.

HRMS *m/z* (ESI): calcd. $C_{10}H_9NO_2$ ($[M + H]^+$): 176.0712; found: 176.0700.

3-(4-Methoxyphenyl)-2*H*-azirine (2b)

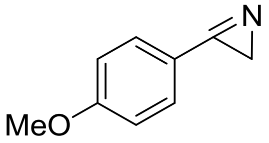

Compound **2b** was prepared in 90% yield (39 mg, 0.27 mmol) from the corresponding vinyl azide as a light yellow oil.

^{1}H NMR (400 MHz, $CDCl_3$): δ = 7.85 (d, *J* = 8.8 Hz, 2H), 7.06 (d, *J* = 8.8 Hz, 2H), 3.90 (s, 3H), 1.74 (s, 2H).

^{13}C NMR (100 MHz, $CDCl_3$): δ = 164.4, 163.3, 131.6, 119.8, 114.6, 55.5, 19.4.

HRMS *m/z* (ESI): calcd. $C_9H_{10}NO^+$ ($[M + H]^+$): 148.0762; found: 148.1123.

All data were in accordance with published data [S5].

3-(4-(*tert*-butyl)phenyl)-2*H*-azirine (2d)

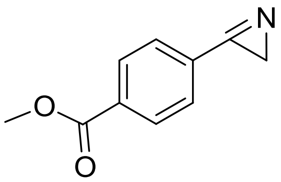

Compound **2d** was prepared in 92% yield (23 mg, 0.13 mmol) from the corresponding vinyl azide as a yellow oil.

^{1}H NMR (400 MHz, $CDCl_3$): δ = 7.85 (d, *J* = 8.5 Hz, 2H), 7.58 (d, *J* = 8.5 Hz, 2H), 1.78 (s, 2H), 1.39 (s, 9H).

^{13}C NMR (100 MHz, $CDCl_3$): δ = 165.4, 156.7, 129.5, 126.1, 125.8, 31.1, 29.7, 19.4.

HRMS *m/z* (ESI): calcd. $C_{12}H_{16}N^+$ ($[M + H]^+$):174.1283; found: 174.1251.

(3-Phenyl-2*H*-azirin-2-yl)methanol (2g)

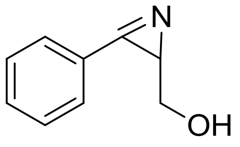

Compound **2g** was prepared in 95% yield (16 mg, 0.11 mmol) from the corresponding vinyl azide as a yellow solid.

MP: 56 °C (MP: 57–58 °C [S6])

^{1}H NMR (400 MHz, $CDCl_3$): δ = 7.91-7.87 (m, 2H), 7.61-7.52 (m, 3H), 3.99 (dd, *J* = 2.6 12.3 Hz, 1H), 3.71 (dd, *J* = 5.0 12.3 Hz, 1H), 2.48 (dd, *J* = 2.6 5.0 Hz, 1H), 1.7 (bs, 1H).

^{13}C NMR (100 MHz, $CDCl_3$): δ = 169.2, 133.2, 129.6, 129.2, 125.3, 64.3, 33.4.

HRMS *m/z* (ESI): calcd. $C_9H_{10}NO^+$ ($[M + H]^+$):148.0762; found:148.0750.

All data were in accordance with published data [S6].

5. General procedure for the photoinduced dipolar cycloaddition of nitrile ylides under flow conditions

The photo reactor (volume: 5.5 mL; details see above) was connected to a syringe pump and at the outlet a collection flask was located. A syringe (ø 13.5 mm) was filled with a solution of the respective vinyl azide **1** (0.05 mmol, 1 equiv) and the

dipolarophile **4** (0.5 mmol, 10 equiv) in CH_3CN (1 mL) and the solution was pumped through the reactor (flow rate: 0.05 mL/min). Washing with CH_3CN was continued until no product was detected at the outlet (monitored by TLC). After evaporation of the solvent, the crude product was purified (if necessary) by column chromatography (petroleum ether/ethyl acetate).

Methyl 4-(3-cyano-3,4-dihydro-2*H*-pyrrol-5-yl)benzoate (5a)

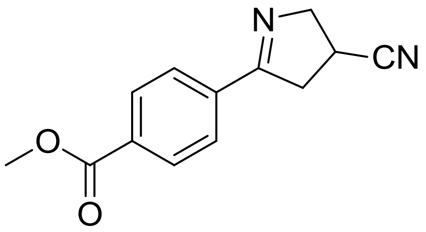

Compound **5a** was prepared in 96% yield (10.9 mg, 0.047 mmol) as a pale yellow solid.

MP: 120.8 °C

^{1}H NMR (400 MHz, $CDCl_3$): δ = 8.12 (d, *J* = 8.4 Hz, 2H), 7.88 (d, *J* = 8.4 Hz, 2H), 4.59-4.51 (m, 1H), 4.45-4.36 (m, 1H), 3.96 (s, 3H), 3.53-3.31 (m, 3H).

^{13}C NMR (100 MHz, $CDCl_3$): δ = 170.1, 166.3, 136.6, 132.4, 129.9, 127.7, 121.1, 65.2, 52.4, 40.4, 25.8.

HRMS *m/z* (ESI): calcd. $C_{13}H_{12}N_2O_2$ ($[M + H]^+$): 229.0977; found: 229.0975.

5-(4-Methoxyphenyl)-3,4-dihydro-2*H*-pyrrole-3-carbonitrile (5b)

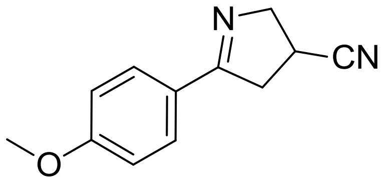

Compound **5b** was prepared in 64% yield (6.4 mg, 0.032 mmol) as a waxy pale yellow solid.

^{1}H NMR (400 MHz, $CDCl_3$): δ = 7.77(d, *J* = 8.8 Hz, 2H), 6.96 (d, *J* = 8.8 Hz, 2H), 4.53-4.44 (m, 1H), 4.38-4.30 (m, 1H), 3.87 (s, 3H), 3.48-3.26 (m, 3H).

^{13}C NMR (100 MHz, $CDCl_3$): δ = 170.0, 162.0, 129.4, 125.6, 121.4, 114.0, 64.9, 55.4, 40.2, 25.8.

HRMS *m/z* (ESI): calcd. $C_{12}H_{12}N_2O$ ($[M + H]^+$): 201.1028; found: 201.1026.

5-(Naphthalen-2-yl)-3,4-dihydro-2*H*-pyrrole-3-carbonitrile (5c)

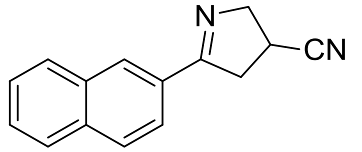

Compound **5c** was prepared in 83% yield (8.3 mg, 0.037 mmol) as a pale yellow solid.

MP: 143°C (MP: 143–144 °C [S7])

^{1}H NMR (400 MHz, $CDCl_3$): δ = δ = 8.13 (s, 1H), 8.06 (dd, *J* = 8.6, 1.7 Hz, 1H), 7.94-7.87 (m, 3H), 7.59-7.55 (m, 2H), 4.58 (dd, *J* = 16.6, 8.8 Hz, 1H), 4.43 (ddt, *J* = 16.6,

8.8 Hz, 1H), 3.64-3.34 (m, 3H). All data were in accordance with published data [S6].

^{13}C NMR (100 MHz, $CDCl_3$): δ = 170.8, 134.7, 132.8, 130.4, 128.7, 128.6, 127.8, 127.6, 126.7, 124.1, 121.3, 65.1, 40.3, 25.8.

HRMS *m/z* (ESI): calcd. $C_{15}H_{12}N_2$ ([M + H]$^+$): 221.1079; found: 221.1075.

5-[5-(4-(*tert*-butyl)phenyl]-3,4-dihydro-2*H*-pyrrole-3-carbonitrile (5d)

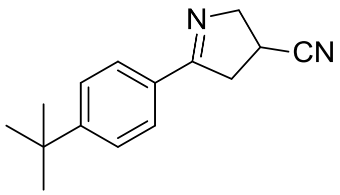

Compound **5d** was prepared in 49% yield (5.5 mg, 0.024 mmol) as a pale yellow oil.

^{1}H NMR (400 MHz, $CDCl_3$): δ = 7.75 (d, *J* = 8.5 Hz, 2H), 7.48 (d, *J* = 8.5 Hz, 2H), 4.56-4.32 (m, 2H), 3.50-3.26 (m, 3H), 1.36 (s, 3H).

^{13}C NMR (100 MHz, $CDCl_3$): δ = 170.5, 154.8, 130.1, 127.5, 125.6, 121.4, 65.0, 40.3, 34.9, 31.1, 25.8.

HRMS *m/z* (ESI): calcd. $C_{15}H_{18}N_2$ ([M + H]$^+$): 227.1548; found: 227.1543.

5-(Pyridin-3-yl)-3,4-dihydro-2*H*-pyrrole-3-carbonitrile (5e)

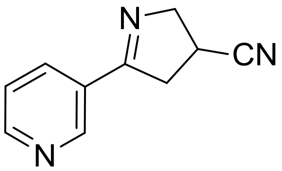

Compound **5e** was prepared in 35% yield (2.9 mg, 0.017 mmol) as a yellow oil.

^{1}H NMR (400 MHz, $CDCl_3$): δ = 8.76(d, *J* = 6.0 Hz, 2H), 7.66 (d, *J* = 6.0 Hz, 2H), 4.62-4.52 (m, 1H), 4.49-4.39 (m, 1H), 3.51-3.32 (m, 3H).

^{13}C NMR (100 MHz, $CDCl_3$): δ = 169.4, 150.7, 147.5, 121.5, 120.8, 65.5, 40.1, 25.8.

HRMS *m/z* (ESI): calcd. $C_{10}H_9N_3$ ($[M + H]^+$): 172.0875; found: 172.0873.

5-Phenyl-3,4-dihydro-2*H*-pyrrole-3-carbonitrile (5f)

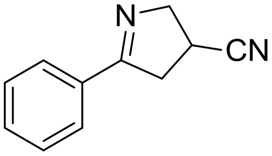

Compound **5f** was prepared in 71% yield (6.1 mg, 0.035 mmol) as a colourless solid.

MP: 95 °C (MP: 95–96 °C [S8])

^{1}H NMR (400 MHz, $CDCl_3$): δ = 7.82 (d, *J* = 7.7 Hz, 2H), 7.56-7.41 (m, 3H), 4.57-4.48 (m, 1H), 4.42-4.33 (m, 1H), 3.53-3.26 (m, 3H).

^{13}**C NMR** (100 MHz, $CDCl_3$): δ = 170.8, 132.8, 131.3, 128.7, 127.6, 121.3, 65.0, 40.3, 25.7.

HRMS *m/z* (ESI): calcd. $C_{11}H_{10}N_2$ ($[M + H]^+$): 171.0922; found: 171.0926.
All data were in accordance with published data [S8].

tert-Butyl 5-[4-(methoxycarbonyl)phenyl]-3,4-dihydro-2*H*-pyrrole-3-carboxylate (5g)

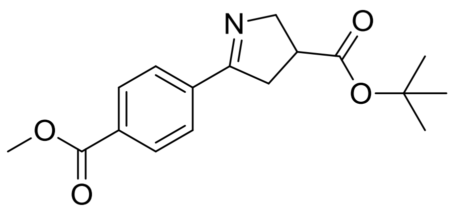

Compound **5g** was prepared in 80% yield (12 mg, 0.039 mmol) as a waxy pale yellow solid.

^{1}**H NMR** (400 MHz, $CDCl_3$): δ = 8.10 (d, *J* = 8.7 Hz, 2H), 7.91 (d, *J* = 8.7 Hz, 2H), 4.47-4.39 (m, 1H), 4.29-4.17 (m, 1H), 3.95 (s, 3H), 3.43-3.15 (m, 3H), 1.49 (s, 9H).

^{13}**C NMR** (100 MHz, $CDCl_3$): δ = 173.5, 171.2, 166.6, 137.8, 131.8, 129.7, 127.6, 81.1, 65.2, 52.2, 42.6, 38.6, 28.0.

HRMS *m/z* (ESI): calcd. $C_{17}H_{21}NO_4$ ($[M + H]^+$): 304.1549; found: 304.1544.

Methyl 4-[3-(morpholine-4-carbonyl)-3,4-dihydro-2*H*-pyrrol-5-yl]benzoate (5h)

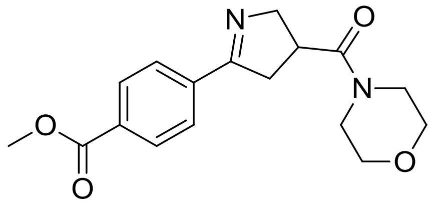

Compound **5h** was prepared in 78% yield (12.5 mg, 0.041 mmol) as a pale yellow oil.

^{1}H NMR (400 MHz, $CDCl_3$): δ = 8.10 (d, *J* = 8.5 Hz, 2H), 7.91 (d, *J* = 8.5 Hz, 2H), 4.49-4.40 (m, 1H), 4.26-4.16 (m, 1H), 3.95 (s, 3H), 3.79-3.50 (m, 10H), 3.23-3.14 (m, 1H).

^{13}C NMR (100 MHz, $CDCl_3$): δ = 171.9, 171.6, 166.6, 137.8, 131.8, 129.7, 127.6, 66.9, 66.7, 65.3, 52.2, 46.2, 42.5, 39.3, 38.9.

HRMS *m/z* (ESI): calcd. $C_{17}H_{20}N_2O_4$ ($[M + H]^+$): 317.1501; found: 317.1499.

Methyl 4-(5-benzyl-4,6-dioxo-3,3a,4,5,6,6a-hexahydropyrrolo[3,4-*c*]pyrrol-1-yl)benzoate (5i)

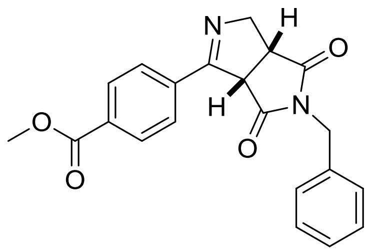

Compound **5i** was prepared in 70% yield (12.7 mg, 0.035 mmol) as a pale yellow solid.

MP: 148.1 °C

^{1}H NMR (400 MHz, $CDCl_3$): δ = 8.22 (d, *J* = 8.5 Hz, 2H), 8.13 (d, *J* = 8.5 Hz, 2H), 7.35-7.29 (m, 5H), 4.75-4.48 (m, 5H), 3.96 (s, 3H), 3.75 (dt, J_1 = 9.0, J_2 = 4.0 Hz, 1H).

^{13}C NMR (100 MHz, $CDCl_3$): δ = 177.2, 172.8, 166.4, 165.8, 135.7, 135.2, 132.4, 129.6, 129.2, 128.8, 128.7, 128.1, 63.5, 56.1, 52.3, 44.5, 42.8.

HRMS *m/z* (ESI): calcd. $C_{21}H_{18}N_2O_4$ ($[M + H]^+$): 363.1345; found: 363.1346.

Di*iso*propyl 5-(naphthalen-2-yl)-3*H*-1,2,4-triazole-1,2-dicarboxylate (5j)

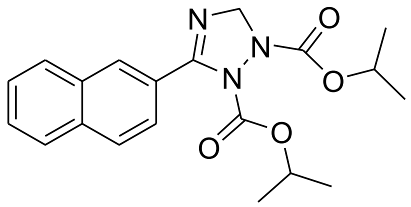

Compound **5j** was prepared in 71% yield (13.3 mg, 0.035 mmol) as a waxy pale yellow solid.

^{1}H NMR (400 MHz, $CDCl_3$): δ = 8.36 (s, 1H), 7.96-7.86 (m, 4H), 7.61-7.52 (m, 2H), 5.43 (brs, 2H), 5.11 (q, *J* = 6.2 Hz, 1H), 4.94 (q, *J* = 6.2 Hz, 1H), 1.38 (d, *J* = 6.2 Hz, 3H), 1.13 (d, *J* = 6.2 Hz, 3H).

^{13}C NMR (100 MHz, $CDCl_3$): δ = 158.5, 157.5, 152.6, 134.7, 132.4, 130.1, 128.9, 127.7, 127.6, 127.5, 126.7, 126.6, 125.8, 73.6, 71.7, 71.1, 22.0, 21.5.

HRMS *m/z* (ESI): calcd. $C_{20}H_{23}N_3O_4$ ($[M + H]^+$): 370.1767; found: 370.1563.

Dimethyl 2-[4-(methoxycarbonyl)phenyl]-1*H*-pyrrole-3,4-dicarboxylate (5k)

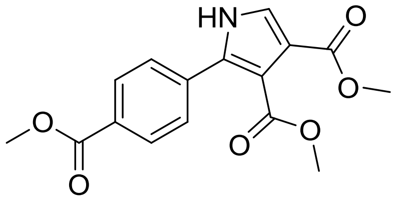

Compound **5k** was prepared in 26% yield (4.1 mg, 0.013 mmol) as a waxy pale yellow solid.

^{1}H NMR (400 MHz, $CDCl_3$): δ = 8.76 (brs, 1H), 8.09 (d, *J* = 8.6 Hz, 2H), 7.56 (d, *J* = 8.6 Hz, 2H), 7.46 (d, *J* = 2.7 Hz, 1H), 3.95 (s, 3H), 3.86 (s, 3H), 3.85 (s, 3H).

^{13}C NMR (100 MHz, $CDCl_3$): δ = 166.4, 163.7, 134.9, 130.1 126.9, 124.3, 52.3, 52.2, 51.6.

HRMS *m/z* (ESI): calcd. $C_{16}H_{15}NO_6$ ($[M + Na]^+$): 340.0797; found: 340.0801.

2-Phenyl-2,5-dihydrooxazole (9)

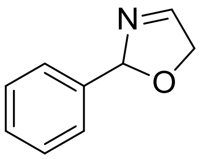

The photo reactor (volume: 5.5 mL, Teflon (FEP) tubing, inner diameter: 0.75 mm) was connected to a syringe pump and, at the outlet side, to a collection flask. A syringe (ø 13.5 mm) was filled with a solution of vinyl azide **1g** (*c* = 0.01 M) in benzene and the solution was pumped through the reactor (flow rate 0.02 mL/min).

Washing with benzene was continued until no product was detected at the outlet (monitored by TLC). After evaporating of the solvent, the crude product was purified by column chromatography (petroleum ether/ethyl acetate = 9/1) and **9** was obtained as a yellow solid (76%).

^{1}H NMR (400 MHz, $CDCl_3$): δ = 8.01 (d, *J* = 6.9 Hz, 2H), 7.58-7.42 (m, 4H), 6.00 (dd, J_1 = 6.9 Hz, J_2 = 4.3 Hz, 1H), 4.52-4.35 (m, 2H).

^{13}C NMR (100 MHz, $CDCl_3$): δ = 167.0, 132.1, 128.7, 128.5, 127.3, 127.0, 90.0, 74.3.

HRMS *m/z* (ESI): calcd. C_9H_9NO ($[M + H]^+$): 148.0762; found: 148.0841.

References

[S1] Wang, Y.-F.; Toh, K. K.; Chiba, S.; Narasaka, K. *Org. Lett.* **2008**, *10*, 5019-5022.

[S2] Jordan, D. *J. Org. Chem.* **1989**, *54*, 3584-3587.

[S3] Kupracz, L.; Hartwig, J.; Wegner, J.; Ceylan, S. *Beilstein J. Org. Chem.* **2011**, *7*, 1441-1448.

[S4] Wang, Y.-F.; Toh, K. K.; Lee, J.-Y.; Chiba, S. *Angew. Chem. Int. Ed.* **2011**, *50*, 5927-5931.

[S5] Hortmann, A. G.; Robertson, D. A.; Gillard, B. K. *J. Org. Chem.* **1972**, *37*, 322-324.

[S6] Sakai, T.; Kawabata, I.; Kishimoto, T.; Ema, T.; Utaka, M. *J. Org. Chem.* **1997**, *62*, 4906-4907.

[S7] Padwa, A.; Dharan, M.; Smolanoff, J.; Wetmore Jr., S. I. *J. Am. Chem. Soc.* **1973**, *95*, 1945-1954.

[S8] Padwa, A.; Gasdaska, J. R.; Haffmanns, G.; Rebello, H. *J. Org. Chem.* **1987**, *52*, 1027-1035.
